# Sketching T cell atlases in the single‐cell era: challenges and recommendations

**DOI:** 10.1111/imcb.70040

**Published:** 2025-06-29

**Authors:** Itana Bojović, António GG Sousa, Sini Junttila, Laura L Elo

**Affiliations:** ^1^ Turku Bioscience Centre University of Turku and Åbo Akademi University Turku Finland; ^2^ InFLAMES Research Flagship Centre University of Turku Turku Finland; ^3^ Institute of Biomedicine University of Turku Turku Finland

**Keywords:** atlas, integration, multi‐omics, single‐cell, T cells

## Abstract

Recent advances in single‐cell technologies have enabled the creation of comprehensive cell atlases, reference maps of various cell types within organisms. Here we specifically focus on T cell atlases, which offer a detailed catalog of the adaptive immune system at single‐cell resolution. As such, they capture cellular diversity, functional states, and spatial dynamics across tissues, developmental stages, and disease conditions. Given the central role of T cells in orchestrating immune responses, their dysregulation underpins autoimmune disorders, cancer progression and failed immunotherapies. Therefore, a unified T cell atlas is critical for decoding such disease mechanisms, identifying therapeutic targets, and advancing personalized treatments. In this article, we explore the latest advances in T cell atlases, describing breakthroughs in multi‐omics technologies, spatial profiling and computational frameworks that resolve transcriptional, epigenetic and proteomic heterogeneity. We also address persistent challenges and highlight strategies to address these gaps. Finally, we discuss emerging frontiers set to reshape our understanding of T cell dynamics in both health and diseases. Together, these insights underscore the transformative potential of T cell atlases in reconstructing precision immunology and accelerating therapeutic innovation.

## Graphical abstract

Recent advances in single‐cell technologies have enabled the creation of T cell atlases, providing detailed maps of adaptive immunity across tissues, development and disease states. These atlases help uncover T cell diversity, functional states and disease mechanisms, aiding in the identification of therapeutic targets. This review highlights key breakthroughs in multi‐omics, spatial profiling and computational analysis while addressing challenges and future directions for leveraging T cell atlases in immunology.

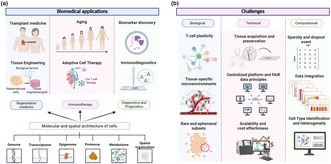

## INTRODUCTION

T cells or lymphocytes are key players of the adaptive immune system, providing cell‐mediated immunity against harmful pathogens or cancer cells. They continuously monitor the human body to maintain immune homeostasis, but dysregulated T cells may fail to target and kill malignant cells and may even trigger an autoimmune response that harms the body's own tissues. This thin balance makes the phenotypic characterization of different T cell populations of utmost importance to understand human health and advance immunotherapies. In recent years, the expansion of single‐cell technologies, which profile molecular features at the single‐cell level in an unprecedented way, has revealed an overlooked diversity of T cell populations and functional states shaped by development, tissue‐specific functions and immune activation. This suggests that more remains to be discovered as technologies continue to improve and studies scale up.

**Figure 1:**
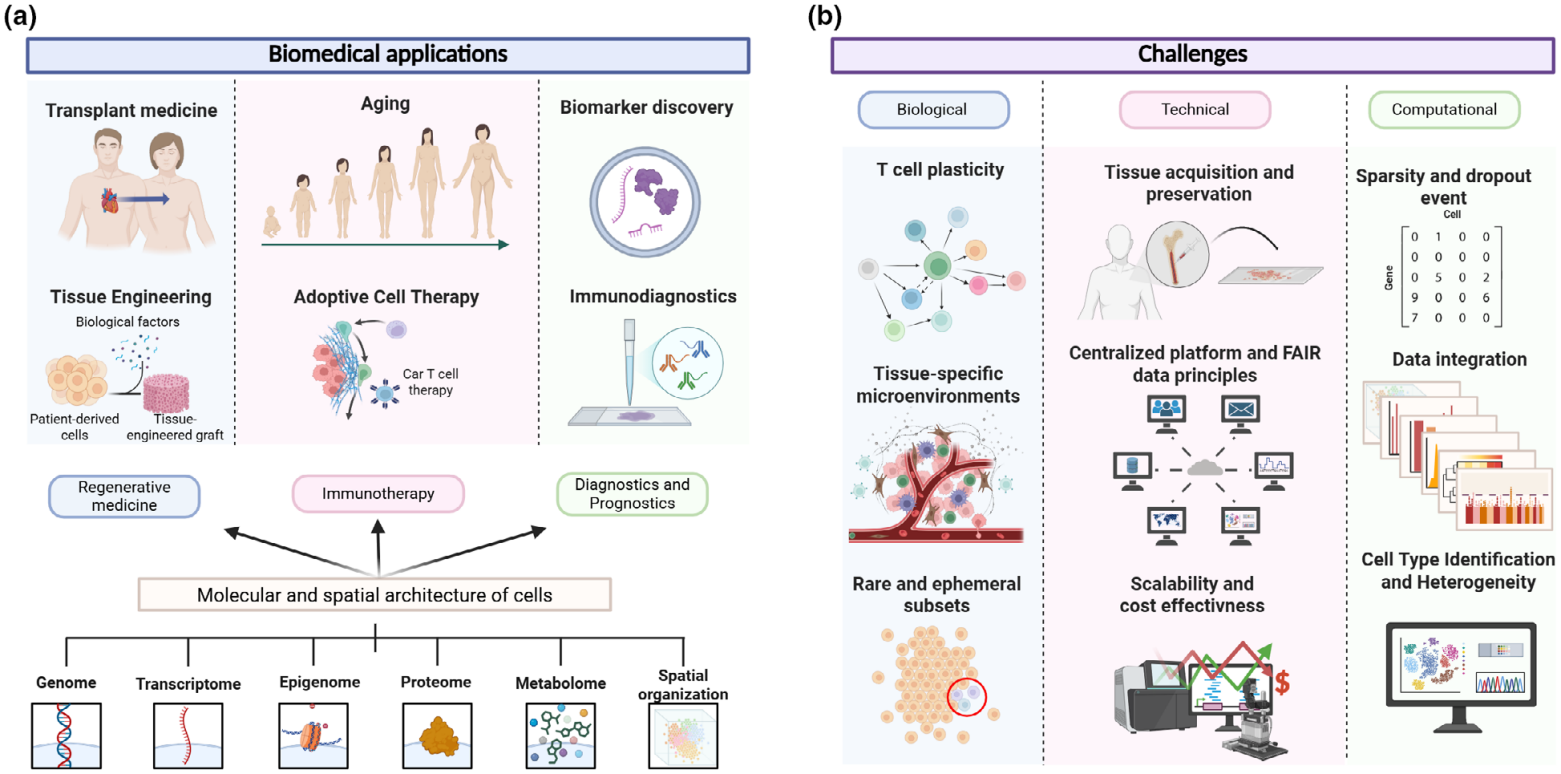
T cell atlas: applications and challenges. **(a)** Examples of biomedical applications enabled by profiling the molecular and spatial architecture of T cells in tissues. **(b)** Challenges encountered in building a T cell atlas. This figure was created with Biorender.com.

The functional plasticity of T cell subsets, such as the one observed in T helper cell populations—Th1, Th2, Th17, Tfh, Treg—highlights the need to catalog their various types and functional states to better understand their roles in immunity. This can be achieved by constructing comprehensive T cell atlases, mapping cells through development^1^ and across multiple tissues.^2^ Such atlases enable the identification of phenotypically relevant cell states and their associated differential expression programs, providing a unique resource for studying disease mechanisms and developing therapeutic strategies^3^ (Figure 1a). This approach can enhance the identification of precise therapeutic targets and establish a foundation for evaluating drug effects. In addition, T cell atlases serve as a tool to monitor and infer immune compatibility in tissue engineering and cell therapy.^4^ Similarly, they can provide insights into transplant medicine by identifying specific T cell populations influencing the body's response to transplanted organs or tissues.^5^ They also can improve our understanding of the immunology of aging and provide insights into the enhancement of immune responses in older adults.^6^

The expansion in T cell atlasing has been driven by breakthroughs in single‐cell RNA sequencing (scRNA‐seq) and subsequently in other single‐cell omics methods. These developments have been underpinned by innovations in technology and computational analysis.^7^, ^8^ Simultaneously, research efforts are converging on integrating scRNA‐seq with a range of omics methods.^9^ Accordingly, pairing scRNA‐seq with other omics strategies allows the examination of genomic, epigenomic and protein‐level information. This integrative approach gives a holistic systems‐level view of individual cells, potentially revealing the interaction of different molecular layers that define cell types and states. This is particularly important given the challenges in accurately delineating cell states from transcriptomics data due to the high degree of transcriptional plasticity in cells. Additionally, single‐cell transcriptomics can be coupled with spatial technologies and offers a view into cellular localization and intercellular interactions within tissue environments.^10^

As single‐cell techniques continue to evolve, researchers can now better explain cellular integrity at both individual and spatial levels. As a result, it is possible to determine cell type, state, transition, location, lineage, as well as cell–cell interactions. Additionally, further advances are paving the way for more accurate predictions of cellular perturbations and their impact on cell fate. Despite this potential, significant biological, technical and computational challenges persist (Figure 1b). Nevertheless, declining costs and rapid advances in computational methods are improving both accessibility and precision. Therefore, it remains to be seen how these advancements will enhance the detection and characterization of rare and small T cell populations, which are one of the key challenges in the creation of a global T cell atlas.

In this article, we review the current state and existing gaps in human T cell atlases in health and disease. In addition, we provide recommendations and describe the opportunities and challenges for building the next generation of T cell atlases.

## CELL ATLASING — CHALLENGES AND RECOMMENDATIONS

Cell atlasing is the comprehensive cataloging of cellular heterogeneity within a defined space and time for a specific biological system of interest.^11^ This process involves exhaustive sampling of the biological system to increase the likelihood of capturing all cell types and states, including rare populations. The success of this Herculean task depends on the effective integration of multiple single‐cell datasets from several donors, which are often obtained using different sampling, preservation, library preparation and sequencing protocols.^11^ The main steps in building a cell atlas are (1) defining the scope, (2) data collection, (3) data integration, (4) quality assessment, (5) annotation and (6) data sharing. The three broad challenges faced in building a T cell atlas are the underlying biological complexity of T cells, the technological limitations of current methodologies, and sufficient computing resources (Figure 1b).

### Defining the scope

Ideally, the scope should not be too broad, to avoid strong or nested batch effects that could mask subtle biological signals, nor too narrow, to ensure comprehensive characterization of cellular phenotypes within the biological system under study. Defining the scope involves selecting the cell type(s) (phenotypic heterogeneity), tissue(s) (space) and developmental phase(s) (time) from the biological system of interest (organism level) under steady (healthy) and/or perturbed (disease) conditions. For example, the cell atlas built by Park *et al*.^1^ cataloged thymic cells across the human lifespan.

### Data collection

The collection of datasets is guided by the defined scope and may rely solely on newly generated data,^2^ publicly available datasets, or both.^1^ Generating new data allows for the standardization of protocols, minimizing technical variation. Although incorporating public datasets avoids cumbersome repetition and potentially enriches the atlas by increasing coverage (e.g. donors and cell heterogeneity) and including otherwise inaccessible samples, it also presents several challenges. Raw data are often unavailable—especially in human studies—limiting use to processed data. Datasets annotated with older reference genome versions can lead to “misalignment” when compared with newer versions or may lack annotations for relevant features, such as long non‐coding RNAs.^12^ Quality control steps may have removed underrepresented populations, such as double‐positive T cells, flagged as doublets.

Single‐cell sequencing technologies used across datasets may vary significantly, which can greatly impact downstream analyses. Key differences between plate‐based (low‐throughput, high‐coverage) and droplet‐based (high‐throughput, low‐coverage) methods influence cell and transcript capture as well as dropout rates.^13^, ^14^ Although immune cells from blood do not require tissue dissociation, immune cells from other tissues do, in order to obtain a viable cell suspension. The cell suspension protocol should be standardized to minimize artifacts arising from differences in cell viability and cell type composition.^15^ Additionally, some samples from problematic tissues may only be available through single‐nucleus RNA sequencing (snRNA‐seq), which lacks cytoplasmic transcripts such as mitochondrial RNAs.^13^ While snRNA‐seq is unaffected by cell size and avoids stress‐related gene expression artifacts caused by dissociation,^13^ it presents challenges when integrating with scRNA‐seq.

Datasets available in single‐cell repositories (e.g. GEO, Single Cell Portal) are not always provided in the most flexible format—raw counts. Instead, they are often transformed (e.g. Transcripts Per Million), which can hinder the downstream application of count‐dependent computational methods, such as Scrublet^16^ or scDblFinder^17^ for doublet detection or scVI for integration.^18^ Metadata associated with public datasets may be missing, aggregated, or incomplete, often requiring harmonization across datasets.^11^ All these factors should be carefully considered for each potential dataset, as they will significantly influence downstream steps, particularly integration.

#### Examples of data modalities

**Figure 2:**
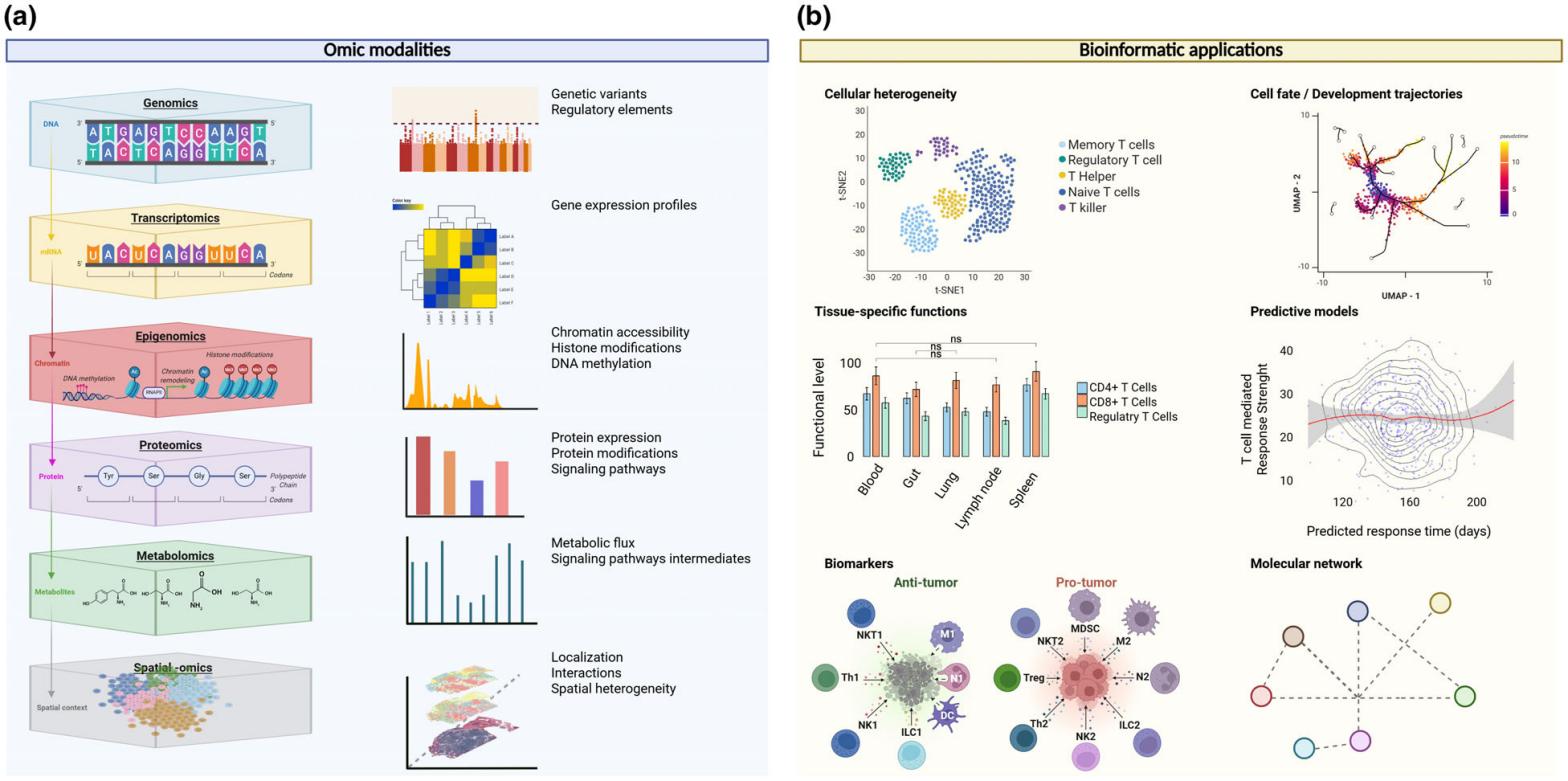
Biological insights provided by different **(a)** omics and **(b)** bioinformatics analyses. This figure was created with Biorender.com.

While single‐cell transcriptomics remains the cornerstone for cell atlas construction, integrating complementary modalities can substantially enhance their biological resolution and functional utility (Figure 2a). A comprehensive cell architecture must describe several interconnected features to accurately capture the full complexity of T cells. These include cellular function, structural characteristics, developmental and differentiation status, interactions with other cells, metabolic profiles and regulatory mechanisms (Figure 2b). Capturing these features often relies on a combination of advanced technologies and rigorous data analysis approaches, where key bioinformatics steps critically shape the outcomes (Table 1).

**Table 1:** Key bioinformatics steps, considerations, challenges and methods for assembling a cell atlas.

Category	Consideration	Challenges	Methods	References
Quality control	Initial QC steps to ensure high‐quality data	Data loss from threshold‐based QC	Adaptive QC models, data‐driven thresholding	19
Contamination	Ambient molecules (RNA, DNA, proteins) from dying cells, debris, or reagents	Background noise	Prior knowledge of negative cell markers, statistical frameworks for background noise reduction	
Sparsity & dropout handling	Differentiate biological from technical zeros in gene and protein expression data	High sparsity in expression matrices	Imputation, non‐imputation (i.e., binarization), pseudo‐bulk analysis	20, 21
Normalization	Mitigate bias due to technical variability (sequencing depth, capture efficiency, library size, among others)	High batch effect variability across platforms	Scaling, normalization, generalized linear models, mixed methods, deep learning‐based methods	22
Feature selection	Selecting most relevant features reduces dimension and redundancy, thus enhance performance in predictive models in downstream analyses	Managing high feature numbers, computational scaling	Filters (statistical criteria), wrappers (predictive models), embedded methods (model training), hybrid and deep learning‐based approaches	23
Dimensionality reduction	Revealing meaningful patterns, improving clustering accuracy and facilitate data visualization	Loss of information, interpretability, scalability	Linear, nonlinear, matrix factorization, graph‐based, probabilistic, deep learning‐based methods	24
Batch correction/data integration	Cross platform and cross‐dataset integration	Harmonizing data, batch effects, technical noise	Global, linear embedding, graph‐based and deep learning‐based models	

For detailed descriptions of available methods and best practices in single‐cell analysis, consult the following online manuals: “Orchestrating single‐cell analysis with Bioconductor”^25^ (https://bioconductor.org/books/release/OSCA) and “Single‐cell best practices”^26^ (https://www.sc‐best‐practices.org).

Cell surface markers provide complementary information for identification of cell types, as conventional short‐read scRNA‐seq alone is sometimes insufficient to resolve the full heterogeneity of T cell subsets.^27^ Cellular Indexing of Transcriptomes and Epitopes by sequencing (CITE‐seq) allows the simultaneous measurement of gene expression and surface markers within the same cell and has been applied to overcome this limitation.^28^, ^29^ T cells possess unique T cell receptors (TCRs) that enable recognition of various antigens and act as intrinsic signatures for detecting clonal expansion, diversity and migration. Integrating TCR‐seq with scRNA‐seq reveals how TCR variability shapes the functional and physiological landscape of T cell populations. However, the vast majority of TCRs are unique to each individual across time and are rarely shared^30^ making it challenging to extract common immune response signatures from TCR‐seq data, thus hampering its utility in T cell atlases.

Given the substantial costs of library preparation and sequencing, single‐cell genomics remains an infrequent choice for single‐cell atlases. As it surpasses transcriptomics in sensitivity for detecting genetic variants, the best results are achieved when they are used in combination,^31^ enhancing the sensitivity for identifying point mutations and structural variations, thus enabling the identification of rare subpopulations and detailed lineage mapping. While this has been particularly impactful in cancer research—revealing evolutionary trajectories of primary tumors and the tumor microenvironment^32^, ^33^—it also has direct relevance to T cell atlases. Specifically, incorporating single‐cell genomics can uncover pathogenic T cell clones, resolve clonotype diversity, and map antigen‐driven T cell evolution in both health and disease, thus enriching T cell atlases with clinically relevant immune heterogeneity.^34^

Single‐cell proteomics directly measures protein abundance, captures post‐translational modifications, reveals protein–protein interactions, and construes functional states and spatial contexts that cannot be properly derived from transcriptomic data. Over the last years, single‐cell proteomics has advanced rapidly; nevertheless, significant challenges remain in achieving comprehensive detection. A central limitation stems from the inherently low abundance of proteins in the cells. Unlike nucleic acids, they cannot be amplified, rendering their detection and quantification particularly demanding. Therefore, highly sensitive instrumentation and proper sample preparation are needed, along with standardized workflows.

Epigenomic profiling reveals key regulatory regions, including non‐coding elements such as enhancers. These modifications are integral to T cell differentiation and the regulation of cell‐type specific gene expression.^35^ For instance, single‐cell epigenomic profiling has revealed alterations in cis‐ and trans‐regulatory elements that orchestrate gene expression in specific T cell subsets.^36^ Transcriptional and epigenetic modifications collectively define attractor states within the cellular landscape, corresponding to distinct cell types. Thus, it is no surprise that epigenomics is one of the most used complementary approaches to single‐cell transcriptomics in atlasing.

Metabolites have a key role in immune response acting as critical activation, differentiation and epigenetic remodeling regulators.^37^ Single‐cell metabolomics is arguably the most complex omics technology, despite being one of the fastest and most cost‐effective.^38^ This complexity arises from the dynamic nature of the metabolome, which is highly susceptible to perturbations at every stage, beginning with sample collection that often disrupts the native state.^38^ Furthermore, external factors such as nutrition, environment and circadian rhythms can significantly alter the innate metabolomic profiles.^39^ In addition, identification and quantification of metabolites have long been significant challenges in metabolomics, largely due to chemical diversity and structural complexity but also the lack of technology for amplification, which restricts the detection of low‐abundance metabolites.^40^ However, ongoing efforts to expand and refine metabolite databases are addressing these limitations.^41^, ^42^, ^43^

Spatial contextualization that encompasses direct measurement of molecular parameters within intact tissue holds great potential by preserving spatial context and cellular interactions. It can be integrated with different omics technologies, enabling a multi‐modal understanding of molecular processes. Among the omics approaches, spatial transcriptomics and spatial proteomics have seen the most widespread adoption. Further exploration of spatial metabolomics could provide valuable insights, particularly given the profound influence of the native microenvironment on cellular biochemistry, especially within the tumor microenvironment.^38^, ^44^ These methods still need to overcome challenges of cost, trade‐offs between sensitivity, throughput and resolution (e.g. lack of true single‐cell resolution), as well as imaging complexity and complex data analysis workflows.^10^ To address some of these limitations, bioinformatic approaches have been developed to integrate spatial information with scRNA‐seq data, enabling the prediction of spatial distributions of undetected transcripts and improving cell type deconvolution.^45^ Despite these advances, the application of spatial single‐cell technologies to T cell atlasing remains limited, particularly because commonly used sample types such as blood and non‐mucosal tissues lack spatial organization and contain relatively few T cells. Therefore, this article focuses on non‐spatial single‐cell technologies, with a particular emphasis on transcriptomics. If the atlas being constructed includes multi‐modal data, such as antibody‐derived tags (e.g. from CITE‐seq) or TCR repertoires, these modalities should be consistently considered across all stages of the atlas construction process: (1) defining the scope, (2) data collection, (3) data integration, (4) quality assessment, (5) annotation and (6) data sharing.

### Data integration

Integration is the key step in cell atlasing. Integration is a broad concept used to refer to the identification of shared cell populations and states across heterogeneously generated datasets. For example, “integration” has been used to describe the identification of shared cell populations within the same modality across different biological conditions (e.g. healthy versus diseased) and/or batches (e.g. sequencing runs), or across modalities of single‐cell data (e.g. RNA‐seq and ATAC‐seq).^46^ Integrating cells across the same modality involves specifying a batch covariate, that is, a variable representing non‐biological experimental confounding effects, such as sample processing or library preparation protocols. A biological variable may instead be provided as the batch covariate, such as species or disease status, when performing cross‐species or cross‐tissue integration tasks.^11^ For example, the cross‐tissue immune cell atlas integrated ~330 K cells by donor (*n* = 12; Dominguez Conde *et al.* ^2^), with each donor contributing multiple tissue samples processed through three distinct protocols, and libraries prepared with or without hashtags using different chemistries. In contrast, the pan‐cancer tumor‐infiltrating T cell atlas integrated ~400 K cells from 316 donors by dataset (*n* = 35; Zheng *et al.* ^47^), each dataset representing a unique combination of independent study (*n* = 22), cancer type (*n* = 21), and library preparation method (*n* = 4). In addition to the batch covariate, some integration methods, such as scVI,^18^ allow the “correction” for other covariates (e.g. age or sex). If cell type annotations are available, these can be used to guide integration by using semi‐supervised integration methods such as scANVI^48^ or STACAS.^49^ Ultimately, a key consideration when choosing an integration method is the reusability of its output model, allowing for reference mapping of additional studies once the model is built. For example, scArches relies on a conditional autoencoder‐based model,^50^ while Coralysis uses logistic regression models.^51^

### Quality assessment

Assessing integration is fundamental to ensure that integration neither distorted the data by masking subtle biological variation nor left technical noise uncorrected. Approaches to assess the quality of integration include low‐dimensional reduction techniques, expression of canonical cell type markers, expression of cluster markers, pre‐ versus post‐integration clustering comparison, automatic cell type annotation, and quantification of bio‐conservation and batch‐correction metrics. Low‐dimensional representations are useful for visually inspecting the overlap between batches. Drawing strong conclusions should, however, be avoided, as they provide an oversimplified view of the data, being unable to preserve high‐dimensional distances.^52^ Canonical cell type markers can help validate and annotate well‐defined clusters across batches, whereas cluster markers identified through differential expression analysis across clusters aid in validating fine‐grained cell populations that may represent distinct cell states.^46^ Comparison of clusters from individual batches with those from the batch‐corrected data ensures that rare populations—particularly those shared across only a few batches—are preserved during integration. Automatic cell type annotation methods, such as Azimuth,^28^ CellTypist,^2^ or ArchMap,^53^ provide fast and comparable annotations that can help validate integration and guide the annotation step. Finally, metrics quantifying biological conservation and batch correction, such as the cell‐specific mixing score from the CellMixS Bioconductor package^54^ or those implemented in the scib python package,^55^ provide objective measures of the effectiveness of integration.

Given the complexity of the integration task, benchmarking methods is common to identify the one that best balances batch correction and biological conservation. Domínguez Conde *et al*.^2^ selected BBKNN^56^ over scVI^18^ for constructing their human cross‐tissue immune cell atlas, as it demonstrated superior performance. In contrast, Sikkema *et al*.^57^ identified scANVI^48^ as the top performer among 12 tested methods for building their human lung atlas. Filippov *et al*.^58^ validated their aging blood immune atlas, integrated with scVI,^18^ against Harmony,^59^ finding that both methods performed similarly. Community‐driven benchmarking platforms such as Omnibenchmark^60^ and Open Problems^61^ provide useful resources and guidelines for benchmarking integration. If the assessment shows subpar results, a new round of integration should be performed using a different set of parameters or an alternative method.

### Annotation

Annotation consists of identifying the cell type identity of individual cells by means of clustering, automatic annotation, or a combination of these two. Clustering‐based annotation is recommended for poorly characterized biological systems, such as tertiary lymphoid structures, whereas automatic annotation is recommended for well‐known tissues, like blood, for which good reference data exist.

There are three types of approaches for automatic annotation: marker‐based, reference‐based, and a combination of the two. Marker‐based methods, such as Garnett,^62^ scType^63^ or MMoCHi,^29^ rely on predefined cell type markers—either positive or both positive and negative—to annotate cells. They generally provide accurate high‐level annotations, where well‐curated markers are available, but often lack low‐level annotations.^64^ On the contrary, reference‐based methods such as SingleR,^65^ Azimuth,^28^ CellTypist,^2^ and ArchMap^53^ are more flexible, allowing the identification of both cell types and subsets at lower levels, but are more computationally demanding. To overcome this, CellTypist (https://www.celltypist.org), Azimuth (https://azimuth.hubmapconsortium.org) and ArchMap (https://www.archmap.bio) provide web applications allowing users without coding experience to annotate their datasets against available references. In both marker‐ and reference‐based approaches, the user may rely on a pre‐existing list of markers or a reference model, or provide their own list or reference. Since the goal of building an atlas is to comprehensively catalog the cellular landscape within a given scope, identification of fine‐grained cell populations is important, as they may represent rare or overlooked subsets. Therefore, an approach combining clustering with automatic annotation is recommended.^29^, ^58^ Regardless of the approach chosen for cell annotation, classifications must follow best practices in the field, following cell type ontology recommendations^66^ to enable comparison, reuse, and hierarchical classification. Annotation is an iterative process that often requires multiple rounds of classification and assessment to fine‐tune cell type labels. The assessment aims to validate annotations based on marker expression, whether canonical or identified through differential expression analysis. Ultimately, one must be able to isolate all cell types and functional states *in vitro* from the respective tissue samples using the identified markers.

### Data sharing

The process of building a single‐cell atlas culminates in its publication. Publishing the atlas requires sharing the data and accompanying materials: code deposition (e.g. GitHub or GitLab), sharing integrated model (e.g. ICP, variational autoencoder), processed data and metadata deposition (e.g. GEO, HCA Data Portal, custom website) and methodology description (article, repositories). In summary, sharing data should follow the FAIR principles. The published atlas should be easily findable—unique identifier—and accessible by submitting raw and processed data to repositories such as GEO or Single Cell Portal. Processed data from public datasets should be deposited in public platforms like Figshare or Zenodo to obtain a unique digital object identifier. Sharing files solely through institutional or custom websites/servers is not recommended, as there are no guarantees regarding their long‐term availability and security. To ensure interoperability and reusability, the atlas should ideally be shared in a single‐cell object format, such as SingleCellExperiment^25^ or SeuratObject^28^ for R, and anndata^67^ for python. This allows for easy distribution of assays, metadata, embeddings and parameters used to build the atlas in a single instance. Additionally, it is imperative to submit the scripts to common repositories such as GitHub or GitLab. Objects and scripts should be distributed under a license that promotes reuse and ensures accessibility, such as CC‐BY (Creative Commons Attribution) or an open‐source software license (e.g. MIT, GPL). Finally, sharing the atlas through a queryable online platform such as CZ CELLxGENE increases visibility and reusability. A good example following these practices is the human cross‐tissue immune cell atlas.^2^

## CURRENT HUMAN T CELL ATLASES

Existing single‐cell atlases comprehensively profiling T cell populations have focused on either healthy^1^, ^68^, ^69^, ^70^, ^71^ or diseased^47^, ^72^, ^73^, ^74^, ^75^, ^76^, ^77^, ^78^, ^79^, ^80^, ^81^, ^82^, ^83^, ^84^, ^85^, ^86^ samples (Table 2). The disease conditions studied can be broadly categorized into cancer,^47^, ^72^, ^73^, ^74^, ^75^, ^76^, ^77^, ^78^, ^79^, ^80^ infections,^81^, ^82^ and (auto)immune disorders.^83^, ^84^, ^85^, ^86^ Park *et al*. sampled thymic cellular diversity at the gene expression level throughout human life, cataloging more than 50 cell populations, including 12 representing T cell subsets, which comprise conventional (e.g. CD4^+^ and CD8^+^ T cells) and unconventional (e.g. CD8αα and γδT) populations.^1^ This allowed the authors to discover a previously uncharacterized population of GNG4^+^ CD8αα T cells in the thymic medulla and identify a TCRα V‐J bias in CD8^+^ T cells using single‐cell TCR analysis. Extending this work to other lymphoid and non‐lymphoid tissues, Wong *et al*.^68^ and Domínguez Conde *et al*.^2^ assembled human cross‐tissue immune cell atlases, analyzing surface markers with mass cytometry and gene expression with scRNA‐seq. Both studies characterized T cell populations and identified tissue‐specific molecular signatures. The latter study included TCR repertoire data for the T cell compartment comprising 185 806 T cells, categorized into 15 cell types representing different states, allowing the description of clonotype distribution across human tissues. A key difference between these studies is the identification of naive and central memory T cells, which, among other markers, relies on the expression of alternative isoforms of the *PTPRC* gene, resulting in the expression of either CD45RA or CD45RO protein. Since this distinction cannot be reliably quantified using scRNA‐seq, naive and central memory T cell populations appeared grouped in Domínguez‐Conde *et al*.^2^

**Table 2:** Description of published healthy and diseased T cell atlases that include fine‐grained cell populations.

Atlas	References	Tissue	Resolution	Study finding	Analytical methods	Number of cells	Number of donors	Cancer/disease	Data availability
Enhancers across helper T cell diversity	71	Blood	136 (not annotated)	Genetic risk of immune‐mediated diseases	5′ snRNA‐seq 5′ scRNA‐seq CITE‐seq snATAC‐seq + 3′ snRNA‐seq	1 M	56	Healthy	NBDC: hum0350 JGA: JGAS000689 GEA and DDBJ: PRJDB13816
General longitudinal blood immune cell atlas	70	PMBC	41	Age related alterations in PBMCs	scRNA‐seq scTCR‐seq scBCR‐seq Protein feature barcoding	2 M	166	Healthy	Synapse: syn49637038
T cell differentiation atlas	69	PBMC	14	Regulatory elements that control CD8^+^ T cell subset gene expression	RNA‐seq ATAC‐seq CRISPRi	Not applicable	24	Healthy	GEO: GSE179613
General thymus cell atlas	1	Thymus	21	T cell development across lifetime in thymus and bias in human TCR repertoire formation	scRNA‐seq scTCR‐seq	256 K	24	Healthy	ArrayExpress: E‐MTAB‐8581 https://developmentcellatlas.ncl.ac.uk
T cell atlas in healthy tissues	68	8 tissues	75	T cell trafficking and functional markers for tissue specificity	CyTOF	Not indicated	31	Healthy	Not available
General T cell lymphoma atlas	72	Skin	5	TH2‐like programs in malignant T cells, spatial interactions with B cells, and tumor‐supportive microenvironment in cutaneous T cell lymphoma	scRNA‐seq TCR‐seq DNA‐seq Bulk RNA‐seq Spatial transcriptomics	420 K	45	Cutaneous T cell lymphoma	https://collections.cellatlas.io/ctcl
T cell acute lymphoblastic leukemia	73	Bone marrow PBMC	9	Cellular and genetic factors contributing to resistance and relapse in T cell acute lymphoblastic leukemia	scRNA‐seq CITE‐seq scATAC‐seq bulk RNA‐seq	604 K	48	T cell acute lymphoblastic leukemia	dbGaP: phs003432
Pre‐infusion CAR T atlas	74	PBMC	17	Pre‐infusion CAR T cells with high type 2 functionality correlation with long‐term leukemia remission	scRNA‐seq CITE‐seq Multiplexed secretomic assay	696 K	88	Acute lymphoblastic leukemia	GEO: GSE262072
Atlas of CD19 chimeric antigen receptor T cells	75	PBMC	26	Elevated glycolysis gene expression in CAR T cells correlates with complete remission at three months	scRNA‐seq	417 K	59	Relapsed/refractory large B‐cell lymphoma	CellXGene: https://cellxgene.cziscience.com/collections/d9a9760c-e43d-404d-86aa-447c9a1e9120
Pan‐cancer atlas of T cells	76	13 tissues	32	Stress response state across different tumor types	scRNA‐seq Spatial profiling	308 K	324	16 cancer types	https://singlecell.mdanderson.org/TCM/
General breast cancer immune cell atlas	77	Breast	11	Unique T cell‐B cell interaction that fosters a more immunosuppressive profile	scRNA‐seq	118 K (T cell: 93 K)	21	Breast tumors	GEO: GSE31519, GSE176078
General urothelial carcinoma cell atlas	78	Urothelial (urinary tract)	6	Basal upper tract urothelial carcinoma features exhausted T cells interacting with immunosuppressive macrophages, driving immune evasion and affecting therapy response	scRNA‐seq	67 K (T cells: 21 K)	12	Upper tract urothelial carcinoma	GSA‐BDC: HRA001867
General gastric cancer cell atlas	79	Stomach Peripheral blood	20	Tc17‐driven T cell exhaustion and stromal‐immune crosstalk drive immunosuppression in gastric cancer	scRNA‐seq scTCR‐seq scBCR‐seq bulk DNA‐seq bulk RNA‐seq	166.5 K	10	Gastric tumor	GSA‐BDC: HRA000704 OMIX: OMIX001073
Hepatocellular carcinoma multicellular ecosystem atlas	80	Liver Lymph node Portal vein thrombus	10	Early tertiary lymphoid structures enrichment with antitumor central memory T cells	scRNA‐seq	25 K	10	Hepatocellular carcinoma	EGA: EGAC00001001616 GEO: GSE149614
Pan‐cancer atlas of T cells	47	15 tissues	41	Landscape of tumor‐infiltrating T cells (heterogeneity, dynamics and key pathways of CD8^+^ T cells exhaustion)	scRNA‐seq scTCR‐seq	398 K	316	21 cancer types	GSA: PRJCA001702 GEO: GSE156728 http://cancer-pku.cn:3838/PanC_T
Sepsis T cell atlas	81	PBMC	32	Dynamic changes of T cell subsets during sepsis onset and progression	scRNA‐seq	91.6 K	16	Sepsis	GEO: GSE1673639, GSE17545310
HIV immune cell atlas	82	PBMC	8	Analysis of gene signatures associated with immune cell exhaustion during human immunodeficiency virus infection	scRNA‐seq	66.5 K	9	Human immunodeficiency virus	GEO: GSE157829
“TCR‐first” T cell atlas	83	Multi‐tissue	49	The utility of a TCR‐first approach	scRNA‐seq scTCR‐seq	494 K	90	Multi‐disease	Zenodo repository: https://doi.org/10.5281/zenodo.10809382
General skin Immune‐Stromal Atlas	84	Skin	9	Immune and stromal network's link to disrupted epithelial differentiation in atopic dermatitis	scRNA‐seq scVDJ‐seq scATAC‐seq	707 K	19	Scleroderma Atopic dermatitis	GEO: GSE158432, GSE153760, GSE147424 https://developmental.cellatlas.io/diseased-skin
Intrahepatic T cell atlas	85	Liver	13	Tissue‐resident, naive‐like CD4^+^ T cells are poised for TH17 differentiation	scRNA‐seq scTCR‐seq CITE‐seq scATAC‐seq	22 K	11	Primary sclerosing cholangitis	ArrayExpress: E‐MTAB‐10143
Colonic CD8^+^ T cells in ulcerative colitis	86	Colon	14	The dynamic interplay between cell states and their interactions with different epithelial cell subtypes in ulcerative colitis	scRNA‐seq scV(D)J‐seq	8,6 K	6	Inflammatory bowel diseases	GEO: GSE148837, GSE148505
Human cell atlas portal				https://www.humancellatlas.org/					
Single cell atlas				https://www.singlecellatlas.org/					
Human immunology project consortium				https://hipc-dashboard.org/					

Background colors denote study classification: green for healthy tissue, light orange for cancer, blue for infections, and light purple for (auto)immune diseases. Some diseased cell atlases also include healthy cells. “Resolution level” refers to the number of T cell clusters or states reported in the original publications and is not directly comparable across studies. Additionally, the table concludes with integrative resources that aggregate multiple independent cell atlas studies. Databases referenced in these studies are as follows: dbGaP, The database of Genotypes and Phenotypes; DDBJ, DNA Data Bank of Japan; GEA, Genomic Expression Archive; GEO, Gene Expression Omnibus; GSA, Genome Sequence Archive; GSA‐BDC, Genome Sequence Archive in BIG Data Center (Beijing Institute of Genomics); JGA, Japanese Genotype–phenotype Archive; NBDC, National Bioscience Database Center Human Database.

Additionally, several other healthy atlases have been published, comprising circulating T cells with different scopes.^58^, ^69^, ^70^, ^71^ Giles *et al*. built a bulk epigenetic and transcriptional cell atlas of 14 sorted circulating T cell populations, providing higher resolution for CD8^+^ T cell populations (10 populations versus 4 for CD4^+^ T cells).^69^ Among these, a population of PD1^+^CD39^+^ CD8^+^ T cells with an exhausted phenotype was identified as the epigenetically closest to tumor‐infiltrating T cells (TILs). This helped identify cytotoxic and WNT signaling signatures as key differentiators of responders and non‐responders, respectively, in anti‐PD1‐treated melanoma patients. Oguchi *et al*.^71^ constructed a multimodal CD4^+^ T cell atlas integrating gene expression and enhancer activity with chromatin accessibility (snATAC‐seq) for approximately one million cells. This study aimed to investigate immune‐mediated diseases and pinpointed the cell‐type specificity of disease‐associated single nucleotide polymorphisms (SNPs) within the identified bidirectionally transcribed candidate enhancers. Finally, Terekhova *et al*.^70^ and Filippov *et al*.^58^ assembled human atlases of aging peripheral blood mononuclear cells (PBMCs). Terekhova *et al*.^70^ profiled approximately two million PBMCs from 166 healthy donors aged 25–85 years. Only certain T cell subsets (TRAV1‐2^−^ CD8^+^ T cells, MAIT and γδ T cells) showed statistically significant changes over the human lifespan. These results were further validated by Filippov *et al*.,^58^ who found a decrease in CD8^+^ naive T cells and MAIT cells with age.

As T cells play a crucial role in cancer, many cell atlases focusing on T cell heterogeneity have been assembled for cancers such as lymphoma,^72^ T cell acute lymphoblastic leukemia (T‐ALL^73^), triple‐negative breast cancer,^77^ urothelial carcinoma,^78^ and gastric cancer,^79^ as well as cancer therapies like CAR T‐cells^74^, ^75^ (see Table 2). To decipher pan‐cancer gene expression patterns in TILs, Zheng *et al*.^47^ and Chu *et al*.^76^ constructed pan‐cancer T cell atlases comprising 21 and 16 cancer types, respectively, with over 300 donors and more than 300 000 cells each. Through trajectory analyses, the atlases enabled Zheng *et al*.^47^ to identify two developmental pathways leading to T cell exhaustion, one progressing through effector memory T cells and the other through tissue‐resident memory T cells. Similarly, Chu *et al*.^76^ identified an overlooked stressed T cell state characterized by the expression of heat shock genes, which was further validated in histological samples through spatial transcriptomics analyses and found to be associated with a poorer response to immune checkpoint therapy.

Disease focused atlases with a scope beyond cancer include those for primary sclerosing cholangitis,^85^ ulcerative colitis,^86^ and human immunodeficiency virus (HIV).^82^ The transcriptomic atlas from Wang *et al*.^82^ analyzed PBMCs from both healthy and HIV‐infected donors, profiling immune populations, including eight distinct T‐cell subsets. This enabled the identification of an HIV‐specific KLRG1^+^ CD8^+^ T cell population with an exhausted phenotype, which could be restored by blocking the inhibitory receptor KLRG1. In a colonic CD8^+^ T‐cell atlas integrating scRNA‐seq, TCR repertoire analysis and CyTOF data from healthy individuals and ulcerative colitis patients, Corridoni *et al*.^86^ identified an IL‐26 expressing CD8^+^ T cell population with an innate‐like phenotype in ulcerative colitis. This population exhibited anti‐inflammatory properties, attenuating acute colitis in a humanized IL‐26 transgenic mouse model. Similarly, Poch *et al*.^85^ identified a tissue‐resident naive‐like CD4^+^ T cell population, with a propensity to acquire a Th17 polarization state, which was expanded in primary sclerosing cholangitis patients, based on their intrahepatic T‐cell atlas comprising 22 198 transcriptomes.

Studies employing single‐cell methods are often limited by the narrow demographic scope of sampled donors. Beyond the limited availability of healthy samples, there is also a need for geographical, gender and ethnic diversity, since ~90% of human genomic data collected to date comes from individuals of European origin.^87^ For the time being, most published studies only report information regarding the number and age of donors. Overall, existing T cell atlases have proven invaluable by enabling the identification of phenotypically relevant cell states in both health and disease, elucidating developmental trajectories, and exploring disease‐related mechanisms with potential therapeutic applications.

## FUTURE PERSPECTIVES

The future of T cell atlases heavily relies on collaborative work. Advancing our understanding of T cells across diverse tissues and conditions requires pooling expertise, data and technologies. This effort is supported by the continuous development and enhancement of computational tools, which enable the analysis and integration of the vast and complex datasets generated. Deep learning methods have recently proven to be highly effective in single‐cell analysis.^88^ A critical next frontier lies in developing predictive models to simulate cellular behaviors, disease trajectories, and response to environmental changes, drug treatments, or genetic perturbations.

The development of T cell atlases will benefit not only from advances in computational single‐cell analysis methods but also from continuous improvements in technological platforms. For instance, 10x Genomics has recently launched a new chromium product GEM‐X, designed for lower cost and higher cell profiling per run. These advancements also include compatibility with previously inaccessible sample types, such as fresh, frozen, PFA‐fixed tissue, whole blood and FFPE samples. Improvements in sample recovery now allow researchers to work more effectively with limited samples like small biopsies and flow‐sorted cells.

Transcriptome profiling is currently the mainstay of T cell atlases, but technological advances and cost reduction may soon enable routine integration of multiple other omics layers. The shift from descriptive profiling to the functional characterization of cellular states is already underway, and this trend is expected to accelerate with the further development of next‐generation approaches. The commercialization of third‐generation sequencing (Oxford Nanopore Technologies and PacBio single‐molecule real‐time sequencing) holds great promise for improved characterization of genomic regions, including structural variations, repeated regions and long‐range interactions. Furthermore, omics technologies aim to minimize assumptions, improving the accuracy of inferred biological mechanisms. Simultaneous profiling of diverse biomolecules will uncover direct molecular interactions. These advancements promise to enable systems‐level insights into intracellular and extracellular regulatory networks.

T cell states are highly dynamic, and capturing those transitions without cell destruction would allow T cell atlases to collect more detailed, time‐based data. Although advances such as live cell imaging paired with single‐cell RNA‐seq offer glimpses into these dynamics, significant issues persist.^89^, ^90^ There are also some advances in direct visualization of chromatin structure in the cell nucleus^90^, ^91^ but we are about to see the future of live cell imaging that will enable the tracking of chromatin changes relative to transcriptional and morphological transitions.

In the future, we also expect an expansion of T cell atlases across different species for the estimation of evolutionary relationships, including conservation and divergence of T cell function. Comparative analyses of immune repertoires, activation pathways and microenvironmental crosstalk across phylogenies will deepen our understanding of adaptive immunity's evolutionary logic.

## AUTHOR CONTRIBUTIONS

**Itana Bojović:** Conceptualization; visualization; writing – original draft; writing – review and editing. **António GG Sousa:** Conceptualization; writing – original draft; writing – review and editing. **Sini Junttila:** Conceptualization; supervision; writing – review and editing. **Laura L Elo:** Conceptualization; funding acquisition; supervision; writing – review and editing.

## CONFLICT OF INTEREST

The authors declare no conflict of interest.
